# Residents’ Cognition, Attitudes, and Intentions to Participate in Long-Term Care Insurance: Moderating Effect of Policy Support

**DOI:** 10.3390/bs14100895

**Published:** 2024-10-02

**Authors:** Zhaohui Sun, Yifan Li, Shaokui Gao

**Affiliations:** Department of Law and Political Science, North China Electric Power University, Baoding 071003, China; jxltxszh@163.com (Z.S.); 18937437693@163.com (Y.L.)

**Keywords:** long-term care insurance, intention to participate in long-term care insurance, policy support, structural equation model

## Abstract

This study took a comprehensive approach to considering residents’ demands and investigated the intentions of residents in Hebei Province to participate in LTCI, exploring the potential for individual participation. By integrating the unique Chinese context and existing literature, this study established a theoretical framework for research hypotheses concerning the influencing factors of residents’ participation intentions. Leveraging a sample of 516 survey responses, we employed structural equation modeling (SEM) and hierarchical regression analysis (HRA) for validation. The research revealed that the heterogeneity of resident attributes has a significant influence on their participation intentions, and a gap exists between residents’ attitudes and intentions, leading to a scenario where attitudes are high but intentions are low. Moreover, perceived usefulness, risk perception, and perceived trust were found to directly affect residents’ intentions and could indirectly affect intentions through the mediating effect of attitudes. Additionally, the moderating role of policy support is instrumental in facilitating the translation of attitudes into actual intentions, bridging the gap between attitudes and participation intentions. These findings will assist researchers in gaining a deeper understanding of Chinese residents’ participation intentions and their underlying influencing factors, ultimately providing a solid foundation for government policy formulation and insurance companies’ strategic adjustments.

## 1. Introduction

China officially entered an aging society in the 21st century. According to the data from the Fifth National Population Census conducted in 2000, the proportions of the population aged 60 and above and aged 65 and above accounted for 10.5% and 7.1% of the total population, respectively, both surpassing the criteria for an aging society. In the Seventh National Population Census conducted in 2020, these proportions increased to 18.7% and 13.5%, respectively, indicating a further intensification of China’s aging population. According to the data on China in the World Population Prospects (2022) dataset released by the United Nations, it is projected that by 2035, the proportion of the population aged 60 and above in China will surpass 30%, marking its entrance into a stage of severe aging. With the continuous intensification of population aging, the issue of disability has gradually become more prominent. According to the data from the Seventh National Population Census, there were approximately 33.66 million disabled elderly individuals in China in 2020, with a disability rate of 12.75% among the elderly population. The China Elderly Health Report released in 2024 indicated that the current size of the disabled population aged 60 and above in China has reached 46.54 million, with a disability rate of 16.2%. It is estimated that by 2050, the number of disabled elderly individuals in China will reach 85.09 million. The growing group of disabled, elderly people has triggered considerable long-term care needs.

While the demand for long-term care continues to rise due to population aging and disability, the traditional family support function in China is continuously weakening, making the elderly’s demand for socialized care increasingly urgent. Furthermore, China’s current social security system cannot address the severe issue of elderly care, making long-term care insurance (LTCI) the key to solving the elderly care problem. In June 2016, the Ministry of Human Resources and Social Security of China issued the “Guiding Opinions on Piloting the Long-Term Care Insurance System,” identifying 15 cities as pilot cities, marking the initiation of national-level promotion of the construction and development of a universal LTCI system. In September 2020, based on the original pilot cities and two key liaison provinces, Jilin and Shandong, China added 14 more pilot cities. According to data from the Chinese Central Government’s official web portal, 49 pilot cities in China are currently implementing LTCI, with the insured population covering 180 million people and over two million people having received benefits. The above practice shows that LTCI is the key to solving the problem of elderly care, and exploring the factors that affect residents’ participation intentions is conducive to the effectiveness of LTCI. Meanwhile, the theoretical community has also confirmed that cognitive variables, attitudes, and policy support have a significant impact on intentions [1,2].

The 2024 Chinese Government Work Report underscores the urgency of advancing the establishment of an LTCI system, particularly as it transitions from pilot projects to comprehensive implementation. To promote the comprehensive planning of China’s LTCI system, it is necessary to not only address the current “fragmentation” in operation modes and policy items but also pay attention to residents’ awareness and intentions regarding LTCI. As the main participants, residents play a crucial role in the development of LTCI and the comprehensive establishment of an insurance system, and their participation intentions constitute direct motivation for their involvement in insurance development and system construction. Therefore, at the time of comprehensive planning for the LTCI system, this study considers the demands of residents. By investigating the intention of residents in Hebei Province to participate in LTCI and its influencing factors, it aims to explore the potential for individual participation, ultimately providing a solid foundation for government policy formulation and insurance companies’ strategic adjustments and facilitating the comprehensive planning and construction of the LTCI system.

## 2. Literature Review and Hypotheses Development

### 2.1. Sociodemographic Factors

Scholars have extensively explored whether sociodemographic factors affect residents’ participation intentions or behaviors [3,4]. In terms of age, Sloan and Norton (1997) found a significant correlation between age and participation intentions, with older individuals exhibiting a higher probability of participating in LTCI [5]. Regarding gender, McCall et al. (1998) revealed that females demonstrate a higher intention to participate than males [6]. In terms of the number of children, Courbage et al. (2020) utilized data from a 2019 survey in Switzerland and discovered that living with children under 18 years old is the strongest determinant influencing parents’ intentions to decide on LTCI [7]. In terms of education, McCall et al. (1998) discovered that the variable most closely associated with purchasing LTCI is educational level, with respondents possessing a Bachelor’s degree or higher having a 2.53 times higher probability of purchasing it than other groups [6]; Cramer and Jensen (2006) also found a significant positive correlation between educational level and participation intentions [8]. Additionally, in terms of income, most scholars believe that income level impacts participation intentions, with individuals with higher incomes displaying stronger intentions while those with lower incomes have lower intentions [9]. Therefore, when examining the factors influencing residents’ participation intention, sociodemographic factors need to be considered as control variables to obtain more reliable results. This study proposes the following hypothesis:

**H1:**  *Residents with different sociodemographic attributes have varying intentions to participate in LTCI.*

### 2.2. Cognitive Variables

#### 2.2.1. Perceived Usefulness

Perceived usefulness is a core variable in the technology acceptance model (TAM), referring to the extent to which users believe that using a certain tool or system will enhance their work performance [10]. According to the TAM, perceived usefulness can directly influence attitudes and behavioral intentions. Shyr (2024) demonstrated that perceived usefulness positively affects students’ attitudes toward accepting augmented reality in automated systems [11]. Research on the impact of perceived usefulness on behavioral intentions spans a wide range of areas, including online purchasing [12,13], green product consumption [14,15], and tourism product consumption [16,17]. These studies have established a significant positive correlation between perceived usefulness and consumption intentions or behaviors. Additionally, some scholars have applied perceived usefulness to explore insurance participation intentions or behaviors. Tsai (2017), through a survey of dockworkers’ perceptions of accident insurance, revealed that perceived usefulness has a significant positive impact on the intention to purchase accident insurance [1]. Jung and Park (2017) found that perceived usefulness in non-face-to-face services moderates the relationship between transaction speed and insurance purchase intention [18]. Despite the fact that perceived usefulness has not been applied to the study of LTCI participation intentions, scholars have confirmed that perceived usefulness is significantly positively correlated with behavioral intentions [19]. Therefore, the following hypotheses were formulated regarding the relationship between perceived usefulness and residents’ attitudes and intentions toward LTCI participation:

**H2:**  *Residents’ perceived usefulness has a significant positive impact on their attitudes toward LTCI;*

**H3**  *Residents’ perceived usefulness has a significant positive impact on their intentions to participate in LTCI.*

#### 2.2.2. Risk Perception

Risk perception refers to the subjective feelings and intuitive judgments of individuals toward various objective risks in their environment, which is subjective [20]. Some scholars have focused on the impact of risk perception on attitudes. Ram and Chand (2016) applied a structural equation model to demonstrate that drivers’ risk perception has a significant impact on their attitudes toward road safety [21]. Additionally, scholars widely acknowledge that the public’s awareness of long-term care risks and the use of LTCI to mitigate them is weak, which restricts their intentions to participate in such insurance [22,23]. Brown and Finkelstein (2009) pointed out that limited awareness of long-term care expenditure risks constrains the public’s purchase intentions [24]; Shi et al. (2023) predicted the decision-making of registered nurses regarding LTCI and discovered that the most influential factor shaping individuals’ decisions about LTCI is their risk appetite score [25]. Thus, it can be seen that risk perception has a significant impact on attitudes and intentions towards insurance participation. The following hypotheses were formulated:

**H4:**  *Residents’ risk perception has a significant positive impact on their attitudes toward LTCI;*

**H5:**  *Residents’ risk perception has a significant positive impact on their intentions to participate in LTCI.*

#### 2.2.3. Perceived Trust

Trust is a stable expectation of individuals to execute cooperative strategies that are beneficial to themselves, and it has a significant impact on individual attitudes. Chetioui et al. (2010) used partial least squares (PLS) to analyze the data of 378 Moroccan online shoppers and found that the level of trust significantly affected consumers’ attitudes toward online shopping [26]. In studies on the factors influencing intention to participate in LTCI, the degree of trust is also an important variable, mainly reflected in two aspects: trust in the government and relevant regulatory departments [27] and trust in insurance companies [28]. Specifically, the former is related to government-led social LTCI, where residents’ trust in the government affects their participation intentions. Liu et al. (2024), based on data from the China Family Panel Studies, analyzed the impact of internet use on informal workers’ participation in the employee public pension scheme (EPPS), with government trust being an important consideration [27]. The latter is related to commercial LTCI offered by insurance companies, where residents’ trust in insurance companies affects their purchase intentions. Brown et al. (2012), using regression analysis based on survey data from participants aged 50 and above in the RAND American Life Panel, found that the public’s lack of trust in insurance companies is an important factor affecting their purchase of LTCI [28]. Buchori and Harwani’s (2021) case study on PT. China Taiping Insurance Indonesia found that trust in insurance companies and their products also has a significantly positive impact on purchase intentions [29]. Therefore, the following hypotheses about the relationship between perceived trust and residents’ attitudes and intentions to participate in insurance were formulated:

**H6:**  *Residents’ perceived trust has a significant positive impact on their attitudes toward LTCI;*

**H7:**  *Residents’ perceived trust has a significant positive impact on their intentions to participate in LTCI.*

### 2.3. Attitudes toward Long-Term Care Insurance

Attitude refers to an individual’s positive or negative evaluation of performing a certain behavior and is an important predictor of behavioral intention [30]. In this study, attitude refers to residents’ individual approval or disapproval orientation toward participating in LTCI. Scholars have used attitudes to predict insurance participation intentions [31]. For example, Liu et al. (2018) surveyed 130 individuals in the construction insurance industry and found that attitudes toward insurance significantly impacted their intentions [32]. Masud et al. (2021) analyzed survey data from 325 households in Malaysia using a structural equation model and found that attitudes toward life insurance significantly influenced families’ tendencies to purchase it [2].

According to the TAM, attitudes are a mediating variable affecting behavioral intentions. Scholars have introduced the mediating role of attitudes into empirical research [33], but there are few studies exploring the mediating role of attitudes in the insurance field. Tan Zheng (2019) conducted an empirical analysis based on user data from the purchase of internet auto insurance and concluded that personal attitudes have a significant positive impact on purchase intentions, and attitudes play a partial mediating role between perceived usefulness and intentions [34]. In other fields, scholars have paid more attention to the mediating role of attitudes [35]. In the consumer field, Camacho et al. (2020) demonstrated that product attitudes mediate the relationship between xenophobism and intention [36]. In the field of pro-environmental behavior research, Liu et al. (2020) found that assessing environmental attitude mediated the relationship between environmental knowledge and pro-environmental behavior [37]. In summary, scholars generally believe that attitudes are an important predictor of behavioral intentions. Therefore, this study proposes the following hypothesis:

**H8:**  *Residents’ attitudes toward LTCI have a significant positive impact on their intentions to participate in LTCI.*

### 2.4. Policy Support

This study considered policy support as a moderating variable in the transformation from residents’ attitudes to participation intentions. Most current research treats policy directly as an external moderating factor in the relationship between consciousness and behavior, investigating the impact of policy on behavior [6,38]. For example, Liu and Zhang. (2023) conducted a study on insurance policies for individuals aged 65 and above in Australia, and the results indicated that more targeted subsidy programs specifically for low-income elderly individuals would have a greater effect on increasing private health insurance coverage [39]. However, scant research has investigated the impact of policy support on intentions or behaviors [40]. In the field of pro-environmental behavior, research by Huilin et al. (2021) shows that residents’ perception of effective policy support has a significant positive impact on their pro-environmental behavior [41]. In the field of job satisfaction research, Huang et al. (2017) found that when policy support is high, employee satisfaction is also higher [42]. Although research applying policy support to explore insurance participation intentions or behaviors is rare, it is not difficult to discern from the above analysis that policy support significantly impacts intentions. Therefore, this study proposes the following hypothesis:

**H9:**  *Policy support plays a moderating role in the transformation from residents’ attitudes to intentions to participate in LTCI.*

**Figure 1:**
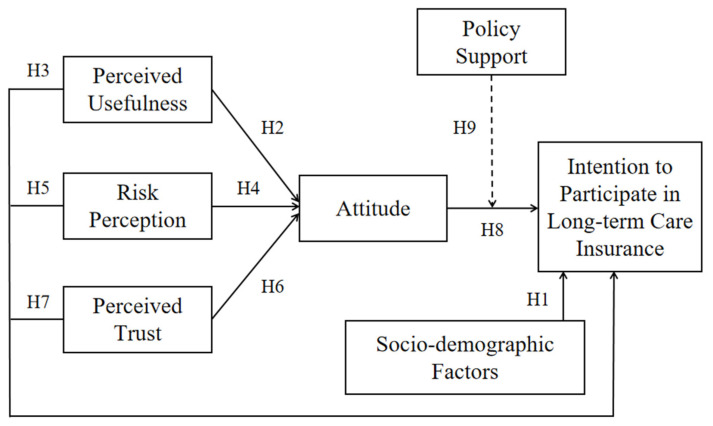
The proposed theoretical framework and research hypotheses.

Overall, the theoretical construction of this model is as follows: guided by behavioral decision theory and based on planned behavior theory, establish a basic model. First, considering the heterogeneity of residents’ participation intentions in social and demographic characteristics and health status, the resident attribute variables were included in the model. According to Ajzen (2011), the Theory of Planned Behavior model is an open explanatory framework that allows adding new variables or path relationships on top of the original model to continuously enhance its explanatory power [43]. This study integrated the perceived usefulness variable from the technology acceptance model [44], the risk cognition variable from the risk theory, and the trust variable from the trust theory and model. These variables were incorporated into the basic model and expanded upon. In terms of path relationship, we chose the attitude with the strongest predictive ability for behavioral intentions in the Theory of Planned Behavior as the mediating variable to examine its mediating role in the path relationship between residents’ psychological cognition and intentions to participate in insurance. In addition, drawing on the attitude-behavior-context theory and the responsible environmental behavior model, the policy support variable was incorporated into the model to examine its moderating effect on the relationship between attitudes and participation intentions. The theoretical framework of this study is proposed in Figure 1.

## 3. Methodology and Measurement

### 3.1. Statistical Analysis Methods

#### 3.1.1. Structural Equation Modeling

Structural equation modeling (SEM) is a widely employed multivariate statistical modeling technique used to test proposed theoretical models and hypotheses [45]. It allows for the concurrent modeling and estimation of intricate relationships among multiple dependent and independent variables, often encompassing concepts that are unobservable and measured indirectly through multiple indicators [46]. In this study, residents’ intentions to participate in LTCI represent an abstract conceptual variable that is challenging to quantify directly, and independent variables such as perceived usefulness and risk perception also exhibit the characteristic of not being directly observable. Based on this analysis, it is evident that structural equation modeling is an appropriate analytical method for this study. This research utilized AMOS 24.0 software for data analysis and hypothesis testing.

SEM comprises two main components: the measurement model and the structural model. The measurement model depicts the relationships between latent variables, whereas the structural model delineates the causal relationships among these latent variables [47]. The basic equation structure of the measurement model is as follows:

y = Λyη + ε x = Λxξ + δ(1)

where y represents endogenous indicators; Λy represents the relationship between endogenous indicators and endogenous latent variables η; x represents exogenous indicators; Λx denotes the relationship between exogenous indicators and exogenous latent variables ξ; ε and δ represent measurement errors on y and x, respectively.

The basic equation structure of the structural model is as follows:

η = Bη + Γξ + ζ(2)

where B represents the relationships between endogenous latent variables, Γ represents the influence of exogenous variables on endogenous variables, and ζ is the residual term in the structural equation.

#### 3.1.2. Hierarchical Regression Analysis

Hierarchical regression analysis (HRA) is commonly used in mediation or moderation studies to explore the relationship between a variable and several independent variables. This method allows researchers to separate different variables into distinct modules, enabling them to observe whether there is a significant change in variance after adding new variables [48]. In hierarchical regression analysis, control, independent, moderate, and interaction variables generated by multiplying centered independent variables and moderator variables are sequentially added to the model. At each step, an equation is formed [49], which is represented as follows:

y = β_0_ + β_1_x_control_ + ε (3)

y = β_0_ + β_1_x_control_ + β_2_x_independent variable_ + ε (4)

y = β_0_ + β_1_x_control_ + β_2_x_independent variable_ + β_3_x_moderator_ + ε (5)

y = β_0_ + β_1_x_control_ + β_2_x_independent variable_ + β_3_x_moderator_ + β_4_x_independent variable_ × x_moderator_ + ε (6)

where x_control_, x_independent variable_, x_moderator_, and x_independent variable×_x_moderator_ represent control, independent, moderator, and interaction variables, respectively, with y being the dependent variable; β_0_ is the intercept term; β_1_, β_2_, β_3_, and β_4_ are regression coefficients; ε is the error term.

### 3.2. Questionnaire Design

This study employed a questionnaire design through three distinct stages. In the first stage, we developed the initial items by leveraging mature scale systems and incorporating selected measurement items from existing research. Subsequently, experts in the field of LTCI were invited to engage in discussions and revisions of the initial items, ultimately resulting in the formulation of an initial questionnaire on residents’ participation intentions. In the second stage, a combination of field and online research was employed for preliminary investigation, and a total of 107 valid questionnaires were collected. Based on a thorough analysis of the research data, the initial questionnaire was further tested and refined. In the third stage, according to the reliability and validity test results of the initial questionnaire, combined with expert consultation and feedback from respondents, the initial questionnaire was comprehensively revised to form the final scale. In this study, relevant variables and their potential structures were measured through multi-item scales, with each item rated on a five-point Likert scale ranging from “strong disagreement” to “strong agreement”, ensuring a comprehensive and nuanced assessment.

**Independent variables.** Cognitive variables were used as independent variables, specifically divided into perceived usefulness, risk perception, and perceived trust. Perceived usefulness was examined through three items: assessing residents’ perceptions of the benefits of participating in LTCI in terms of potential cost or service compensation (PU1), alleviating the human and economic burdens of family care (PU2), and improving the quality of life in old age (PU3), respectively [50]. Risk perception was evaluated through four items: assessing residents’ perceptions of their future risk status (RP1, RP2) and the financial burden of LTCI (RP3, RP4) [22,24], such as “As I enter old age, I may need long-term care” and “If long-term care is needed, the expense would pose a significant economic burden on me and my family.” Perceived trust was measured through four items adapted from studies such as [28], gauging residents’ trust in “The government’s ability to establish a comprehensive LTCI system” (PT1), “The regulatory authorities’ ability to effectively oversee LTCI” (PT2), “Insurance companies’ ability to effectively manage LTCI” (PT3), and “The sustainability of LTCI” (PT4).

**Mediator variable.** Participation attitudes encompassed residents’ attitudes toward LTCI policies and intention to participate in such insurance [31,32]. Two items, “LTCI is an important measure to address population aging” (AT1) and “If a LTCI system is implemented, I support it” (AT2), were used to measure residents’ attitudes toward LTCI policies. The item “I am receptive to participating in LTCI” (AT3) was used to assess residents’ participation intentions.

**Moderator variable.** This section measured the moderating effect of policy support on the transformation of residents’ attitudes to their participation intentions through three items [51,52]. These include three types of policy support: “The government provides subsidies for LTCI participation” (PS1), “The government has implemented adequate guidance and regulatory policies for LTCI” (PS2), and “The government guides insurance companies to develop LTCI that is more closely aligned with residents’ needs” (PS3).

**Dependent variable.** Residents’ intentions to participate in LTCI were measured using items adapted from [53]. Six items were set: “I am willing to participate” (IN1, IN4), “I plan to participate” (IN2, IN5), “I am willing to encourage my family members to participate in social LTCI” (IN3), and “I am willing to purchase commercial LTCI for my family members” (IN6).

### 3.3. Formal Research and Data Collection

#### 3.3.1. Data Collection Process

This study adopted a convenient sampling method and selected Hebei Province as the research location. According to data from the seventh national census, the population aged 60 and above in Hebei Province is 14.81 million, accounting for 19.85% of the total population. This places the province in the stage of moderate aging, with a rate higher than the national average. In May 2016, Julu County was identified as the first pilot county for LTCI within the province. According to data from the Hebei Provincial Medical Security Bureau, by the beginning of 2023, the pilot regions for the provincial LTCI system covered 16.55 million people, with a total of 80,900 individuals having received benefits. Hebei Province has a certain foundation in LTCI, which can prevent respondents’ judgments of their participation intentions from being influenced by a lack of understanding of LTCI. In the implementation of this research, based on the economic development levels of various prefecture-level cities, this study selected Tangshan and Shijiazhuang in economically developed areas, Baoding and Langfang in moderately developed areas, and Hengshui and Chengde in less-developed areas, totaling six prefecture-level cities for questionnaire distribution. These cities exhibit clear levels of development and represent the overall situation of Hebei Province well. In the selection of research participants, this study targeted middle-aged and young residents aged 19–59, who will constitute the elderly population in the future. As the main contributors to LTCI premiums, studying their participation intentions is particularly important. The formal research began in July 2023 and lasted 30 days, with a total of 600 questionnaires distributed, 552 questionnaires collected, and 516 valid questionnaires, representing an effective questionnaire rate of 86%.

#### 3.3.2. Sample Characteristic Analysis

Table 1 displays the demographic information of the respondents.

Hebei Province is a populous region, with individuals aged 0–44 accounting for 72.45% of the total population. This is consistent with the 71.50% proportion of research subjects aged 0–44 in the total sample. Regarding gender, female and male respondents accounted for 52.10% and 47.90% of the sample, respectively, showing a relatively balanced gender distribution, which is consistent with the structure of 50.50% male and 49.50% female in Hebei Province. Respondents with only one child formed the majority, accounting for nearly half of the sample, followed by respondents without children (25.60%). Respondents with more than three children were the least represented, accounting for only 3.30% of the sample. In terms of education, respondents with a Bachelor’s degree made up the largest group (32.00%), followed by those with a high school degree (22.50%), an associate degree (20.20%), a middle school degree or below (15.70%), and a Master’s degree or above (9.70%), showing a decreasing trend. Regarding monthly income, the sample distribution was relatively balanced, with most respondents earning between CNY 3001 and CNY 6000 and the fewest earning more than CNY 10,000.

## 4. Results and Discussion

### 4.1. Descriptive Statistical Analysis

**Figure 2:**
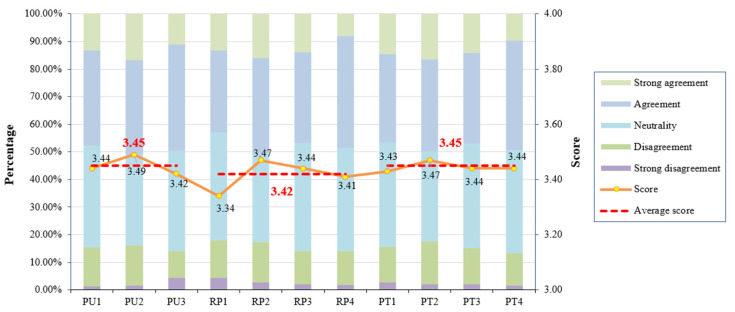
Level of residents’ cognition.

Based on the results presented in Figure 2, the mean scores for the perceived usefulness measurement items PU1-PU3 were 3.44, 3.49, and 3.42, respectively, indicating a generally moderate level of overall perceived usefulness. Among these, residents perceived the strongest usefulness in long-term care insurance’s ability to alleviate the human and economic burdens of home care, which aligns with the reality that many residents choose to participate in LTCI primarily to reduce the pressure borne by their families in providing care. The mean scores of the four items of risk perception were 3.34, 3.47, 3.44, and 3.41, respectively, suggesting that residents have a strong awareness of the severe consequences of long-term care, while their cognition of the likelihood of long-term care risks was the weakest. The mean scores for the four items related to perceived trust were 3.43, 3.47, 3.44, and 3.44, respectively, indicating that residents have a higher level of trust in the regulatory authorities of LTCI while showing similar levels of trust in the other three items.

**Figure 3:**
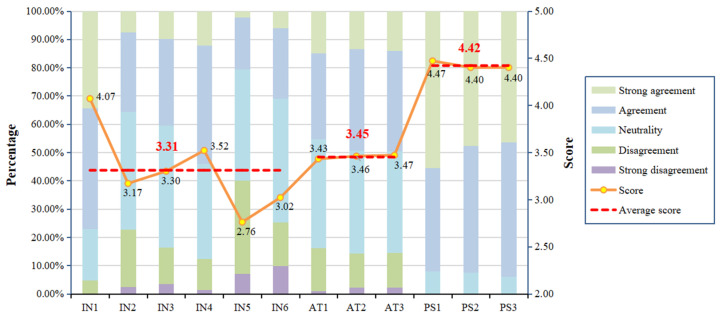
Level of residents’ intentions, attitudes, and policy support.

Figure 3 presents the scores and proportions of residents’ intentions to participate in LTCI. In terms of the mean scores for each measurement item, IN1, IN4, and IN3 followed in sequence, with scores of 4.07, 3.52, and 3.30, respectively. For these measurement items, 4.90%, 12.40%, and 16.50% of residents, respectively, provided scores of 1 or 2, indicating that the overall level of residents’ participation intentions was not high, highlighting the urgent need to explore the influencing factors and their mechanisms affecting residents’ participation intentions. The mean scores for the three measurement items related to attitudes toward participation were 3.43, 3.46, and 3.47, respectively, indicating that residents hold consistent attitudes across these items. In terms of the proportion of options, residents who provided scores of 4 or 5 for AT1–AT3 accounted for 45.30%, 49.30%, and 49.50%, respectively. The proportions of other options were also similar. Furthermore, it can be seen that the attitude scores were higher than the intention scores (3.45 > 3.31), indicating that attitudes have not fully translated into intentions. This phenomenon is referred to in the literature as the attitude–intention gap [54]. In the actual survey, the effectiveness of government policy support perceived by residents was used as a metric to evaluate policy support. The mean scores for the measurement items PS1, PS2, and PS3 were 4.47, 4.40, and 4.40, respectively, indicating that residents perceive government subsidies for participation as the most effective, while the other two items were the same.

### 4.2. SEM Analyses

#### 4.2.1. Measurement Model Testing

AMOS 24.0 software was used to conduct confirmatory factor analysis (CFA) on the formal survey data to determine the adequacy of the measurement model. The model fit indices were as follows: *χ^2^*/df = 2.089 ≤ 5, GFI = 0.931 ≥ 0.8, AGFI = 0.912 ≥ 0.8, RMSEA = 0.046 ≤ 0.08, CFI = 0.982 ≥ 0.9, NFI = 0.967 ≥ 0.9, TLI = 0.979 ≥ 0.9, and IFI = 0.982 ≥ 0.9 [55]. All of these fit indices were within the recommended range, indicating that the measurement model aligned well with the data. Based on composite reliability (CR) and Cronbach’s alpha coefficient (*α*), the reliability of each construct was tested, with the results shown in Table 2. The CR values were calculated using standardized factor loading coefficients (*λ*) and error variances (*θ*), and the results showed that the CR values ranged between 0.85 and 0.95, significantly higher than the standard value of 0.7, indicating that the composite reliability passed the test. The *α* values for each variable ranged between 0.85 and 0.94, significantly higher than the standard threshold of 0.7 [56], indicating good consistency among the measurement items within each variable and good reliability of the scale.

Convergent validity was used to test the construct validity of the scale. According to the confirmatory factor analysis results in Table 2, the *λ* values of the measurement items were all greater than the standard value of 0.5 [57], and all were statistically significant (*p* < 0.001), indicating good convergent validity for each variable. Furthermore, the average variance extracted (AVE) of each variable ranged from 0.65 to 0.74, all greater than 0.5, which again demonstrates that each variable had good validity.

#### 4.2.2. Structural Model Testing

This study used AMOS 24.0 software to test the theoretical framework and research hypotheses based on the structural equation model with maximum likelihood estimation. The model fit indices were as follows: *χ*^2^/*df* = 3.061, GFI = 0.887, AGFI = 0.862, RMSEA = 0.063, CFI = 0.957, NFI = 0.938, TLI = 0.952, and IFI = 0.957. Although these indices are acceptable, there is still room for improvement in model fit. Therefore, the model was refined based on MI. The refined model fit indices were as follows: *χ*^2^/*df* = 2.248, GFI = 0.922, AGFI = 0.901, RMSEA = 0.049, CFI = 0.975, NFI = 0.956, TLI = 0.971, and IFI = 0.975, indicating that the overall fit of the model was better. The standardized path coefficients and hypothesis testing results of the theoretical framework are shown in Table 3.

In terms of control variables, age (*β* = 0.055, *t* = 3.789, *p* < 0.01) had a significant positive impact on insurance participation intention. We found, in the interview, that elderly people are more interested in LTCI. As their physical functions decline, they pay more attention to insurance. This discovery is also consistent with Sloan’s research findings [5]. Gender (*β* = 0.031, *t* = 2.209, *p* < 0.05) had a significant negative impact on participation intentions. This conclusion is consistent with the findings of McCall et al. (1998) [6]. Women have a higher average life expectancy than men and thus face a greater risk of needing long-term care. To mitigate this risk, female residents are more inclined to choose to participate in LTCI. Education (*β* = 0.044, *t* = 2.839, *p* < 0.01) also had a significant positive impact on participation intentions. Residents with higher education levels have stronger cognitive abilities regarding risks and are willing to use insurance to mitigate them. At the same time, they have a stronger ability to accept new things, naturally leading to a higher intention to participate in long-term care insurance [8]. The above analysis showed that H1 was supported.

The results in Table 3 also indicate that H2–H8 were all supported. Specifically, perceived usefulness and attitudes (*β* = 0.234, *t* = 12.269, *p* < 0.001), risk perception and attitudes (*β* = 0.423, *t* = 15.987, *p* < 0.001), and perceived trust and attitudes (*β* = 0.379, *t* = 13.479, *p* < 0.001) all showed significant positive correlations, supporting H2, H4, and H6, consistent with the technology acceptance model, risk theory, and trust theory, respectively. The results also reveal that risk perception had a greater direct impact on attitudes than perceived usefulness and perceived trust (0.423 > 0.379 > 0.234). This finding can be explained by the fact that when residents have a higher cognitive ability to perceive risks in the future, their attitudes will be more positive. Furthermore, perceived usefulness and intentions (*β* = 0.204, *t* = 9.125, *p* < 0.001), risk perception and intentions (*β* = 0.208, *t* = 7.055, *p* < 0.001), and perceived trust and intentions (*β* = 0.317, *t* = 9.222, *p* < 0.001) all showed significant positive correlations. This indicates that residents with stronger perceived usefulness, higher risk perception abilities, and higher levels of trust have stronger participation intentions, supporting H3, H5, and H7, respectively. Moreover, perceived trust had a greater impact on participation intentions than the other two factors. This shows that trust in LTCI is more likely to stimulate residents’ intentions to participate. Attitudes had a positive and significant impact on intentions (*β* = 0.228, *t* = 8.939, *p* < 0.001), indicating that more positive attitudes lead to stronger intentions, supporting H8. This confirms the role of attitudes in the theory of planned behavior.

Table 4 shows that perceived usefulness (*β* = 0.053, *p* < 0.001), risk perception (*β* = 0.096, *p* < 0.001), and perceived trust (*β* = 0.086, *p* < 0.001) had significant indirect effects on the intention to participate in LTCI through attitudes. Therefore, attitudes play a partially mediating role in the perceived usefulness/risk perception/perceived trust–intention relationship.

### 4.3. HRA Analyses

This study hypothesized that policy support is a moderating variable in the relationship between attitudes and intentions. The analysis in Section 4.1 already confirmed that there is an attitude–intention gap. This section further investigates whether introducing the variable of policy support can help bridge this gap. An HRA was conducted to test the moderating effect of policy support and H9.

The KMO values for attitudes, intentions, and policy support were 0.74, 0.90, and 0.72, respectively, all exceeding 0.6, indicating that these items can be analyzed through exploratory factor analysis (EFA). Using principal component analysis with varimax rotation to analyze these four variables, it was found that each variable extracts one common factor, with cumulative variance explanation contribution rates of 80.48%, 78.24%, and 77.15%, respectively. Furthermore, the respective α values for the aforementioned items were 0.88, 0.94, and 0.85, significantly higher than 0.7. It is reasonable to group the measurement items within each variable into one factor. In the HRA, the average scores of the measurement items for attitudes, intentions, and policy support were used for empirical testing.

**Figure 4:**
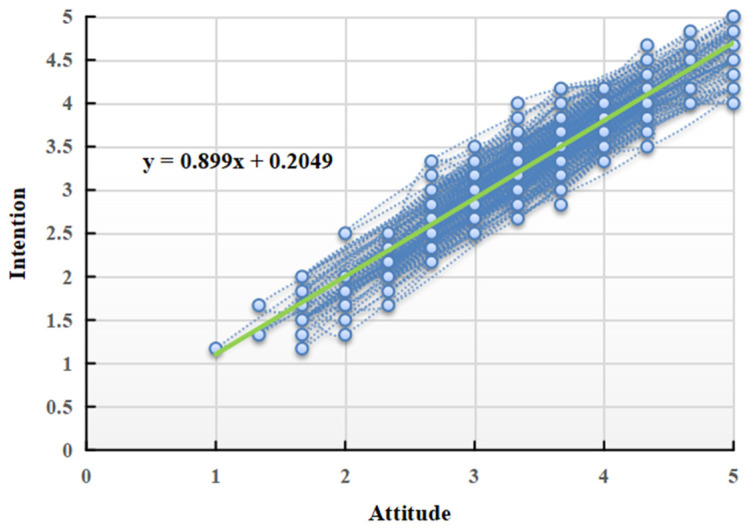
Scatter diagram between attitudes and intentions.

Based on the scatter diagram between attitudes and intentions (as shown in Figure 4), most data points are close to a straight line, indicating a linear correlation. In light of this, a linear ordinary least squares (OLS) regression was applied to test the research questions. Qu et al. (2014) argued that if a variable is significantly correlated with both the independent and dependent variables, this variable may play a moderating role between them [58]. Data analysis revealed that policy support is significantly positively correlated with both attitudes and intentions. It can be preliminarily judged that policy support is a moderating variable.

The study further used HRA to test the moderating effect of policy support on attitudes and intentions. According to the introduction of HRA steps in Section 3.1.2, first, sociodemographic variables were included in the model, followed by attitudes and policy support, and finally, the interaction term between attitudes and policy support was included in the model. The results of the HRA are shown in Table 5.

The control variables explained 35.4% of the variance in intention in Model 1. Among them, age was significantly positively correlated with intentions in Models 1–4, indicating that older residents have a stronger participation intention. Gender was significantly negatively correlated with intention, indicating that female residents have a stronger intention to participate than male residents. This verifies the structural model test results for age and gender, respectively. Model 2, which included control and mediating variables, explained 89% of the variance in intentions. Attitudes were significantly positively correlated with intentions in Model 2 (*β* = 0.888, *t* = 49.953, *p* < 0.001), Model 3 (*β* = 0.888, *t* = 49.477, *p* < 0.001), and Model 4 (*β* = 0.828, *t* = 36.862, *p* < 0.001), indicating that attitudes can be effectively translated into intentions. Model 3, which included control, independent, and moderating variables, explained 89% of the variance in intentions. Model 4 explained 89.4% of the variance in intentions. Furthermore, the interaction term between attitudes and policy support was significantly positively correlated with intentions (*β* = 0.086, *t* = 4.315, *p* < 0.001), indicating a significant moderating effect. The explanatory power of the model continuously increased with the addition of new variables. At the same time, the *F*-values in all four models were significant at the *p* < 0.001 level, and the F change value (Δ*F*) in Model 4 was 18.617, which was significant at the *p* < 0.001 level, further proving that policy support had a significant moderating effect. Additionally, all variance inflation factor (VIF) values were less than 2.5, indicating no multicollinearity among the independent variables in the model. In summary, H9 was supported.

## 5. Policy Implications

By conducting moderate-scale surveys and research to understand the current intentions of residents to participate in LTCI and exploring the factors that influence their intentions, it is beneficial to provide a basis for government policy formulation and strategic adjustments for insurance companies. This enhances the stability and sustainability of the LTCI market and supports the comprehensive and coordinated development of the LTCI system. Based on the results of empirical research, this study proposes policy implications for government and insurance companies that are conducive to enhancing residents’ intentions to participate in insurance.

First, perceived usefulness has a significant positive impact on residents’ participation intentions. Therefore, it is necessary to strengthen publicity and guidance to enhance residents’ perception of the usefulness of LTCI policies. The government needs to carry out publicity work on LTCI so that residents can understand the coverage scope, service content and forms, and relevant regulations of LTCI and form an overall understanding of LTCI among residents. On this basis, a key introduction should be given to the protection functions of LTCI, including functional content such as receiving compensation, reducing family burden, and improving the quality of life in old age through participation, as well as more specific knowledge content such as reimbursement ratios and payment methods at different levels. In addition, different marketing methods should be adopted for different groups of people, families, and regions, with a focus on rural residents with low education levels, so that this group can clearly understand the significance of participating in long-term care insurance. Additionally, insurance companies can hold regular product seminars or explanatory meetings, using live or recorded online broadcasts to provide multi-dimensional and comprehensive explanations of their products, highlight their differences from other health insurance systems, enhance residents’ understanding of LTCI knowledge, and recommend reasonable insurance policies for different groups. Second, the government and insurance companies should work together to make residents aware that long-term care risks are currently more prevalent, and LTCI can effectively transfer this risk. Insurance companies can use both online and traditional media for joint publicity and organize educational and entertaining online or offline activities, distribute risk awareness manuals, and disseminate risk education content to enhance residents’ risk awareness and increase their participation intentions under the guidance of the government. Third, perceived trust has the highest direct impact on the intention to participate in LTCI. Therefore, the government must continuously improve the supervision system and refine the agreement management, as well as the supervision and audit system for nursing service institutions and professionals. The government can also guide social forces to participate in supervision. Social organizations participate and publish professional supervision reports, while individuals rely on information platforms for supervision. The service quality of nursing institutions and nursing staff should be fully examined, and the management department should strictly punish illegal behavior to jointly maintain the standardized operation of the long-term nursing service market. In addition, third-party evaluation agencies can be introduced to conduct comprehensive and multi-level evaluations of insurance institutions on a regular basis. The government should sign contracts with third-party regulatory agencies to separate the actual managers and supervisors of the insurance system through delegation, fully leveraging the independence of the third-party regulatory agencies themselves. In the process of participating in social management, commercial insurance institutions need to formulate corresponding management policies based on the types, products, and channels of participation and strengthen supervision and management of the management process, eliminating irrational price competition and misleading sales behavior to standardize the compliant operation of insurance institutions, strengthen the integrity construction of services, and establish a good image in the industry. Once again, standardized claims standards should be established, and fairness and impartiality should be ensured during the claims process. In addition, insurance institutions need to strengthen the investigation of the insurance demand market and customer satisfaction, analyze the possible impact of insurance services on customers based on their needs and feedback, and form the final service concept. Finally, policy support plays a positive moderating role in insurance participation attitudes and intentions; therefore, the government needs to provide strong policy support and guidance. On the one hand, the government should maintain the sustainability of policies in the design process of LTCI policies so that residents can form stable expectations of future benefits. Additionally, they need to take into account the uneven development between regions, formulate policies flexibly, and ensure the implementation of policies. On the other hand, the government can guide insurance companies to participate in the construction of the LTCI system through policies such as bidding and tendering, providing a useful supplement to social insurance, and promoting the development of commercial LTCI through policies such as tax reductions and exemptions and financial subsidies, adjusting its market share and guiding its stable development.

## 6. Conclusions

This study investigated residents’ intentions to participate in LTCI and their influencing factors by employing the SEM and HRA methods. It also examined the mediating role of participation attitudes and the moderating role of policy support. Finally, policy implications were proposed. The research conclusions are as follows:

First, through descriptive statistical analysis and HRA, it was found that residents with different sociodemographic attributes have varying intentions to participate in LTCI. Second, the results of the descriptive statistics showed that the overall level of residents’ participation intentions is not high, and there is a clear attitude–intention gap, with the level of attitudes outweighing intentions. Third, according to the results of the SEM analysis, cognitive variables (perceived usefulness, risk perception, and perceived trust) were found to have a significant direct impact on residents’ intentions to participate and can indirectly affect intentions through the mediating role of attitudes. Among them, perceived trust has the highest direct effect on participation intentions, indicating that trust in LTCI is more likely to stimulate residents’ intentions to participate. Finally, the results of the HRA showed that policy support has a significant positive moderating effect on the path relationship between attitudes and intentions. The moderating effect of high policy support can strengthen the transformation of attitudes into participation intentions more effectively, bridging the attitude-intention gap that arises during the process of residents’ participation.

Based on the above conclusions, policy implications were proposed from the perspectives of government and insurance institutions to enhance residents’ intentions to participate in insurance and better play the role of LTCI.

It is worth noting that there are still limitations in this study, and further research is needed. First, Hebei Province was selected as the research area. In pursuit of scientific and rigorous research, we divided Hebei Province into three categories: economically developed, moderately economically developed, and economically underdeveloped areas, reflecting the hierarchy of economic development levels. This well represents the overall situation of Hebei Province, but it is not fully representative of the whole of China. In the future, the research scope should be further expanded by selecting corresponding provinces and cities from eastern, central, and western China for research so that the research results can better reflect the reality of China. Second, field research was conducted, and 600 paper questionnaires were distributed. Of these, 516 valid questionnaires were recovered, resulting in a high effective recovery rate. However, the total sample size of the study was slightly insufficient overall. It is necessary to expand the sample size further in conjunction with developing the research scope.

## Figures and Tables

**Table 1:** Description of the sample (N = 516).

Attributes	Category	Frequency	Percentage (%)
Age	1 = 19–30	155	30.00%
	2 = 31–44	214	41.50%
	3 = 45–59	147	28.50%
Gender	0 = female	269	52.10%
	1 = male	247	47.90%
Quantity of children	1 = 0	132	25.60%
	2 = 1	255	49.40%
	3 = 2	112	21.70%
	4 = over 3	17	3.30%
Education	1 = Middle school degree or below	81	15.70%
	2 = High school degree	116	22.50%
	3 = Associate degree	104	20.20%
	4 = Bachelor’s degree	165	32.00%
	5 = Master’s degree or above	50	9.70%
Income (monthly)	1 = ¥3000 or under	99	19.20%
	2 = ¥3001–6000	146	28.30%
	3 = ¥6001–8000	129	25.00%
	4 = ¥8001–10,000	94	18.20%
	5 = More than ¥10,000	48	9.30%

**Table 2:** Reliability and validity test results.

Constructs	Items	*λ*	*θ*	CR	*α*	AVE	KMO
Perceived usefulness	PU1	0.804 ***	0.35	0.85	0.85	0.65	0.73
	PU2	0.803 ***	0.36				
	PU3	0.802 ***	0.36				
Risk perception	RP1	0.817 ***	0.33	0.89	0.89	0.66	0.84
	RP2	0.835 ***	0.30				
	RP3	0.814 ***	0.34				
	RP4	0.783 ***	0.39				
Perceived trust	PT1	0.826 ***	0.32	0.89	0.89	0.66	0.84
	PT2	0.828 ***	0.31				
	PT3	0.829 ***	0.31				
	PT4	0.773 ***	0.40				
Attitude	AT1	0.836 ***	0.30	0.88	0.88	0.71	0.74
	AT2	0.845 ***	0.29				
	AT3	0.843 ***	0.29				
Policy support	PS1	0.762 ***	0.42	0.85	0.85	0.66	0.72
	PS2	0.897 ***	0.20				
	PS3	0.773 ***	0.40				
Intention to participate in LTCI	IN1	0.864 ***	0.25	0.95	0.94	0.74	0.90
	IN2	0.823 ***	0.32				
	IN3	0.884 ***	0.22				
	IN4	0.902 ***	0.19				
	IN5	0.856 ***	0.27				
	IN6	0.837 ***	0.30				

Notes: *λ* is the standardized factor loading coefficient; *θ* is the error variance; CR = composite reliability; AVE = average variance extracted; *α* is Cronbach’s alpha coefficient; *** *p* < 0.001.

**Table 3:** Hypotheses’ test results.

Research Hypothesis	Direction	*β*	*t*-Value	*p*-Value	Result
H1: Perceived usefulness → Attitude	+	0.234	12.269 ***	<0.001	Supported
H2: Perceived usefulness → Intention	+	0.204	9.125 ***	<0.001	Supported
H3: Risk perception → Attitude	+	0.423	15.987 ***	<0.001	Supported
H4: Risk perception → Intention	+	0.208	7.055 ***	<0.001	Supported
H5: Perceived trust → Attitude	+	0.379	13.479 ***	<0.001	Supported
H6: Perceived trust → Intention	+	0.317	9.222 ***	<0.001	Supported
H7: Attitude → Intention	+	0.228	8.939 ***	<0.001	Supported
**Control Variables**					
Age → Intention	+	0.055	3.789 ***	<0.001	Supported
Gender → Intention	−	0.031	2.209 *	0.027	Supported
Quantity of children → Intention	−	0.004	0.259	0.796	Rejected
Education → Intention	+	0.044	2.839 **	0.005	Supported
Income → Intention	+	0.023	1.590	0.112	Rejected

Notes: *β* = standardized path coefficient; *t*-value = CR; * *p* < 0.05, ** *p* < 0.01, *** *p* ≤ 0.001.

**Table 4:** Standardized path coefficients for the mediation effect analysis.

Independent Variables	Mediators	Dependent Variables	Direct Effect	Indirect Effect	Total Effect	Mediating Effect
Perceived usefulness	Attitude	Intention	0.204	0.053 ***	0.257	Part
Risk perception	Attitude	Intention	0.208	0.096 ***	0.304	Part
Perceived trust	Attitude	Intention	0.317	0.086 ***	0.403	Part

Notes: *** *p* ≤ 0.001.

**Table 5:** Analysis results of the moderating effects.

Variable		Dependent Variable			
		Model 1	Model 2	Model 3	Model 4
Control variables	Age	0.412 *** (11.286)	0.064 *** (3.840)	0.064 *** (3.820)	0.062 *** (3.784)
	Gender	−0.169 *** (−4.722)	−0.037 * (−2.482)	−0.037 * (−2.463)	−0.038 * (−2.579)
	Quantity of children	−0.106 ** (−2.853)	−0.024 (−1.525)	−0.024 (−1.515)	−0.027 ^+^(−1.741)
	Education	0.234 *** (5.987)	0.026 (1.566)	0.026 (1.558)	0.022 (1.347)
	Income	0.095 ** (2.572)	0.020 (1.312)	0.020 (1.310)	0.021 (1.410)
Mediator	Attitude		0.888 *** (49.953)	0.888 *** (49.477)	0.828 *** (36.862)
Moderator	Policy support			0.0001 (0.008)	0.019 (1.245)
Interaction term	Attitude × Policy support				0.086 *** (4.315)
*R* ^2^		0.360	0.892	0.892	0.895
Adjusted *R*^2^		0.354	0.890	0.890	0.894
*F*-value		57.382 ***	697.567 ***	596.740 ***	542.582 ***
Δ*R*^2^		0.360	0.532	0.000	0.004
Δ*F*		57.382 ***	2495.281 ***	0.000	18.617 ***
*N*		516	516	516	516

Notes: Standardized regression coefficients are reported in models 1–4; Values in parenthesis are *t*-statistics; *N* = Number of observations; ^+^ *p* < 0.1, * *p* < 0.05, ** *p* < 0.01, *** *p* < 0.001; The values of VIF < 2.5.

## Data Availability

The data that support the findings of this study are available from the corresponding author upon reasonable request.
